# Health-related quality of life and all-cause mortality in patients with diabetes on dialysis

**DOI:** 10.1186/1471-2369-13-78

**Published:** 2012-08-03

**Authors:** Tone Britt Hortemo Østhus, Nanna von der Lippe, Lis Ribu, Tone Rustøen, Torbjørn Leivestad, Toril Dammen, Ingrid Os

**Affiliations:** 1 Department of Nephrology, Oslo University Hospital, Ullevål, N-0484, Oslo, Norway; 2 Institute of Clinical Medicine, Faculty of Medicine, University of Oslo, Oslo, Norway; 3 Faculty, Lovisenberg Diaconal, Oslo University College, Oslo, Norway; 4 Department of Organ Transplantation, Oslo University Hospital, Oslo, Norway; 5 Institute of Basic Medical Sciences, Department of Behavioural Sciences in Medicine, Faculty of Medicine, University of Oslo, Oslo, Norway

**Keywords:** Dialysis, Diabetes, Foot Ulcers, QOL, Mortality

## Abstract

**Background:**

This study tests the hypotheses that health-related quality of life (HRQOL) in prevalent dialysis patients with diabetes is lower than in dialysis patients without diabetes, and is at least as poor as diabetic patients with another severe complication, i.e. foot ulcers. This study also explores the mortality risk associated with diabetes in dialysis patients.

**Methods:**

HRQOL was assessed using the Short Form-36 Health Survey (SF-36), in a cross-sectional study of 301 prevalent dialysis patients (26% with diabetes), and compared with diabetic patients not on dialysis (n = 221), diabetic patients with foot ulcers (n = 127), and a sample of the general population (n = 5903). Mortality risk was assessed using a Kaplan-Meier plot and Cox proportional hazards analysis.

**Results:**

Self-assessed vitality, general and mental health, and physical function were significantly lower in dialysis patients with diabetes than in those without. Vitality (p = 0.011) and general health (p <0.001) was impaired in diabetic patients receiving dialysis compared to diabetic patients with foot ulcers, but other subscales did not differ. Diabetes was a significant predictor for mortality in dialysis patients, with a hazard ratio (HR) of 1.6 (95% CI 1.0-2.5) after adjustment for age, dialysis vintage and coronary artery disease. Mental aspects of HRQOL were an independent predictor of mortality in diabetic patients receiving dialysis after adjusting for age and dialysis vintage (HR 2.2, 95% CI 1.0-5.0).

**Conclusions:**

Physical aspects of HRQOL were perceived very low in dialysis patients with diabetes, and lower than in other dialysis patients and diabetic patients without dialysis. Mental aspects predicted mortality in dialysis patients with diabetes. Increased awareness and measures to assist physical function impairment may be particularly important in diabetes patients on dialysis.

## Background

Diabetic nephropathy has become one of the most frequent causes of end-stage renal disease (ESRD) worldwide, and an increasing number of diabetes patients require haemodialysis or peritoneal dialysis treatment. The incidence of diabetes patients requiring maintenance dialysis has been rising in Norway during the last decade, but is still lower than what has been observed in other parts of Europe and the U.S. [1-4]. In 2009 in Norway, about 18% of patients undergoing dialysis had diabetes as a comorbid condition. Diabetic nephropathy was also the cause of ESRD in 18% of patients undergoing dialysis, and the majority of these patients were type 2 diabetics [4]. The considerable rise in the number of diabetes patients requiring dialysis is likely to increase even further as the population is aging, the prevalence of obesity is increasing, and survival rates after cardiovascular events have improved. Although diabetes patients with ESRD are usually considered for renal transplantation, high comorbidity, especially concomitant cardiovascular diseases, may limit their possibilities for future transplants.

Health-related quality of life (HRQOL) is an important issue for both diabetes patients and dialysis patients [5-7]. HRQOL, and in particular its physical aspects, has been found to be a predictor of mortality in ESRD patients receiving dialysis [8-10]. Self-reported physical aspects of HRQOL are also a predictor for mortality in type 2 diabetes patients without ESRD [11], and, this finding has been extended to include elderly patients with type 2 diabetes [12]. However, the data are scarce and conflicting with regard to how self-perceived physical and mental health may affect survival in diabetes patients on dialysis [13,14]. Few studies have compared HRQOL in dialysis patients with and without diabetes, and those that have provide inconsistent results [6,7,13-15]. Relating HRQOL to various subgroups may provide important clinical information about particularly vulnerable patients. Previously, HRQOL has been assessed in the diabetic population in Norway, and specifically in diabetic patients with foot ulcers [16]. The results indicated that patients with diabetic foot ulcers had a lower HRQOL, especially with regard to the physical aspects of HRQOL, compared to the general diabetic population. However, there have been no comparisons undertaken between diabetes patients with severe complications such as ESRD and those with foot ulcers.

Thus, the objectives of this study were, first, to test the hypothesis that the HRQOL in prevalent dialysis patients with diabetes is lower than in those patients without diabetes, and second, to test the hypothesis that the HRQOL in diabetes patients undergoing dialysis is at least as poor as in diabetes patients with foot ulcers. Finally, we examined the mortality risk associated with diabetes in patients on chronic dialysis.

## Methods

### Study patients and design

The first study objective examined prevalent dialysis patients, and consisted of a cross-sectional cohort and a prospective cohort, with a median follow-up time of 3.6 years. All adult patients (≥18 years) receiving haemodialysis or peritoneal dialysis in 10 different dialysis units (with a catchment area of more than two millions persons) were screened for study participation. They were eligible to be included in the study if they were in stable condition and had received maintenance dialysis for more than 2-months, as previously detailed [17]. Adequate proficiency in the Norwegian language was compulsory. Both oral and written information about the study was provided to the patients, and a signed informed consent was required for enrolment. The enrolment rate was 72.4%, with 301 patients included (close to 1/3 of the prevalent dialysis population in Norway). None of the patients were lost to follow-up. Nurses and physicians were specifically trained in applying the study instruments, which consisted of self-administered questionnaires, as detailed previously [17]. The National Committee for Medical Health Research Ethics in Norway approved the study protocol, and concession was obtained from the National Data Inspectorate. The investigation was conducted according to the Declaration of Helsinki.

The second study objective compared the HRQOL in three groups of diabetes patients: the group of 78 dialysis patients with diabetes, a group of 127 diabetes patients with foot ulcers on or below the malleoli, taken from an anonymised database of patients treated at outpatient clinics in the Oslo area [16], and a group of 221 diabetes patients without complications, who had participated in the Norwegian Survey of Level of Living in 2002. We also had a group of 5903 persons without diabetes from the general population [16]. The study of the diabetes patients with foot ulcers took place five years prior to our cross-sectional study. Data concerning the time of renal transplantation and time of death were retrieved from the Norwegian Renal Registry [4].

### Demographic and clinical data

Demographic and clinical data from the dialysis patients, including age and gender, were collected from reviews of hospital charts and/or direct questioning of the patients. The diagnosis and type of diabetes mellitus was based on information from hospital records and laboratory results. A dialysis patient was considered to have diabetes mellitus if diabetic nephropathy was the primary cause of renal failure, or if diabetes was present as a comorbidity. Comorbidity was assessed using the modified Charlson comorbidity index (CCI, 18) but without age (i.e.*,* not adding a score of 1 for each decade above 40 years). Since both diabetes and diabetic nephropathy were included in the calculation, a separate comorbidity score was also calculated after correcting for diabetes and/or diabetic nephropathy. The demographic and clinical data for the diabetic patients with foot ulcers has been presented previously [16,18], and are therefore summarised only briefly in the results section.

### Assessment of HRQOL

The Kidney Disease and Quality of Life Short Form version 1.3 (KDQOL-SF, 20) was applied to assess generic and disease-specific HRQOL. The first part comprised the Medical Outcome Study, a 36-item Short-Form Health Survey (SF-36) that measures the generic dimensions of HRQOL using eight subscales [19]. The physical summary (PCS) and the mental component summary (MCS) were also calculated according to a scoring algorithm [20]. The kidney disease-specific portion of the KDQOL-SF consists of 43 items classified into 11 specific kidney-related scales [21].

### Statistical analyses

Descriptive data are presented either as mean ± standard deviation (SD), or as medians with interquartile range (IQR). Percentages are given for categorical variables. The Student’s *t*-test or the Mann–Whitney test were used for comparisons between two groups, and the Kruskal-Wallis test or one-way analysis of variance was performed when comparing several groups. Survival was analysed from the time of entry into the study until death, and also after excluding patients with shorter observation period than 2 months. A sensitivity analysis was also done for the mortality risk, examining patients after they had been on dialysis > 4 months. Survival data were not collected from patients after renal transplantation. Cumulative survival curves were constructed using the Kaplan-Meier method, with patients stratified by the presence or absence of diabetes, and with patients stratified for the presence or absence of kidney disease. Survival rates were compared using the log-rank test. Patients were stratified by PCS or MCS scores (above or below median), and survival was analysed using Kaplan-Meier plots. The magnitude of the associations between diabetes and mortality risk were estimated with Cox-proportional hazard models, using a univariate model entering only diabetes as a variable, then adjusting for age, coronary artery disease, duration of dialysis, and either PCS or MSC. This provided a hazard ratio (HR) with a 95% confidence interval (CI). Since diabetes and the comorbidity score were considered substantially correlated (r = 0.63), comorbidity was not included as a co-variable in the model; instead, the presence or absence of coronary artery disease was included as an important clinical variable. A one-way analysis of variance (F-statistics) was used to test differences in the means of the subscales of SF-36 between groups, and thereafter, post hoc multiple comparisons using the Bonferroni test. The data were analysed using SPSS for Windows, version 19 (SPSS, Chicago, U.S.).A value of p ≤ 0.05 was considered significant.

## Results

Clinical characteristics of the dialysis population are provided in Table 1. Dialysis patients without diabetes had a slightly lower body mass index (p =0.013), but otherwise did not differ from diabetes patients in this cohort. Diabetic nephropathy was the cause of renal failure in forty-one (52%) of the diabetes patients undergoing dialysis. The comorbidity scores differed between these groups, with the highest scores in the dialysis patients with diabetes [Table 1]. However, after removing the diabetes and diabetic nephropathy variables, there was no longer a significant difference in comorbidity scores between the two groups (median 3 IQR 2–4 vs. 3, 2–4, mean 3.5 ± 1.5 and 3.3 ± 1.3).

**Table 1:** Clinical characteristics (given as mean [SD], median [IQR], or percentage) in prevalent dialysis patients (n = 301) with and without diabetes mellitus

	**Diabetes mellitus, n = 78**	**No diabetes mellitus, n = 223**
Age years	58.9 (13,5)	60.1 (17.1)
Male gender %	68	65
Smokers	31	25
Married or cohabitant %	63	64
Body mass index kg/m^2^	26.1 (5.5)	24.4 (4.5)
Haemoglobin g/dL	12.4 (1.4)	12.0 (1.5)
Albumin g/L	38.2 (4.3)	38.2 (5.2)
CRP mmol/L	6.6 (2.5-15.1)	6.0 (2.1-12.7)
PTH pmol/L	32.3 (13.4-47.8)	22.9 (13.5-37.9)
Dialysis vintage years	10 (5–24)	10 (6–24)
Peritoneal dialysis %	24	18
Kt/V (haemodialysis)	1.31 (0.19)	1.38 (0.29)
Urea mmol/L	21.8 (5.5)	22.4 (6.8)
*Comorbidity*		
Charlson comorbidity index	5.5 (1.6)	3.3 (1.3)
Nephrosclerosis %	24	37
Previous renal graft %	11	18
Diabetes type 1%	41	
Diabetes type 2%	59	

Diabetic patients with foot ulcers and diabetic dialysis patients did not differ in age (p = 0.3), gender (p = 0.5) or smoking habits (p = 0.9). Body mass index was higher in the diabetic patients with foot ulcers (28.1 ±6.2 kg/m^2^ vs. 26.1 ±5.5 kg/m^2^, p = 0.02). These groups also differed with respect to kidney function, as none of the diabetic patients with foot ulcers had ESRD, 49 % had normal serum creatinine levels, and only 21% had diabetic nephropathy.

There were also significant differences between the diabetic and non-diabetic dialysis patients with respect to walking disability (54 vs. 35%, χ^2^ = 7.9, p = 0.005), but not with respect to the use of walking aids or wheel chairs (31 vs. 25%, not significant [ns]). Three of the diabetic patients had a lower extremity amputation, compared to no amputations in the non-diabetic dialysis patients. A total of seven patients in the dialysis population reported claudication, along with four in the diabetic group and three in the non-diabetic group. The incidence of leg cramps as perceived by the dialysis patients did not differ between the diabetic and non-diabetic groups (49 vs. 46%, ns), nor did the occurrence of restless legs (49 vs. 46%, ns).

### Comparisons of KDQOL-SF-36 subscales between dialysis patients with and without diabetes

Using the generic SF-36 health survey, dialysis patients with diabetes had lower mean scores for mental health (median 68; IQR 65–88 vs. 80; 65–88, p = 0.006), vitality (40; 20–50 vs. 45; 29–65, p = 0.019) and general health (50; 30–60 vs. 40; 20–50, p = 0.01) compared to non-diabetic dialysis patients. The other subscales of the SF-36 health survey did not differ significantly between the groups. There was a significant difference in MCS scores between patients with and without diabetes (46 IQR 38–54 vs. 50 IQR 40–57, p = 0.04), and this difference was of a similar magnitude for the PCS scores (33 IQR 28–43 vs. 37 IQR 31–46, p = 0.051).

Social support was reduced for dialysis patients with diabetes compared to those without diabetes (83; 50–100 vs. 83; 67–100, p = 0.019), while the other kidney-specific quality of life scores did not differ significantly between the dialysis groups, i.e.*,* symptoms (72 vs. 75), effect of kidney disease (66 vs. 69), burden of kidney disease (25 vs. 31), work status (0 vs. 0), cognitive function (87 vs. 93), quality of social interaction (80 vs. 87), sexual functioning (63 vs. 75), sleep (65 vs. 62), staff encouragement (75 vs. 88), and satisfaction with care (66 vs. 69).

### Comparisons of SF-36 subscales between diabetic patients on dialysis and a sample from the general population, and between diabetic patients with and without foot ulcers

**Figure 1:**
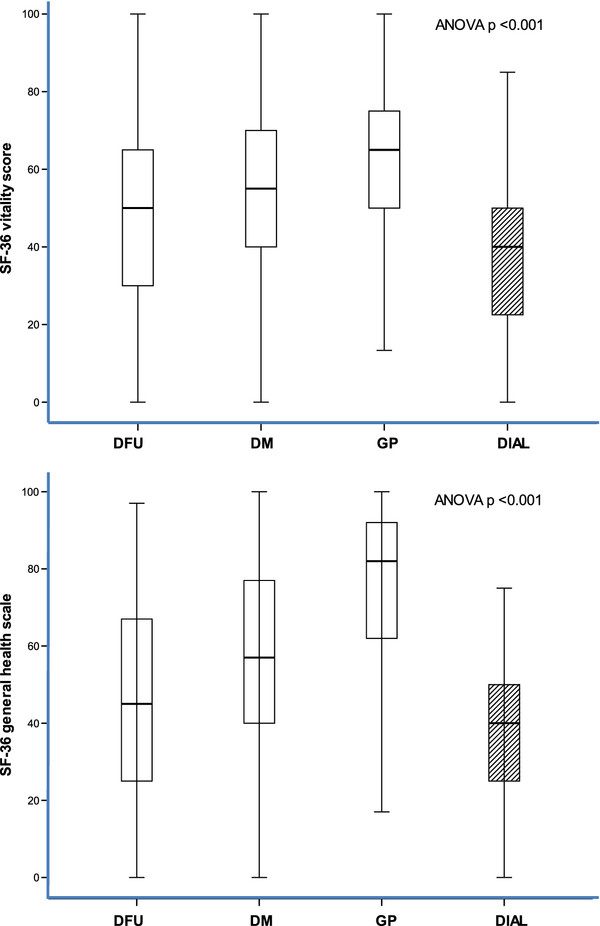
Boxplots of vitality score (upper panel) and general health (lower panel) in diabetes patients on dialysis (DIAL, hatched bar), diabetes patients with foot ulcers (DFU), diabetes patients (DM), and the general population (GP).

Dialysis patients with diabetes had a significantly lower HRQOL than the general population [Table 2]. There were also significant differences between the SF-36 subscale scores, both for mental and physical health, in diabetes patients receiving dialysis compared to diabetes patients who were not receiving dialysis [Table 2]. The SF-36 subscale scores for diabetes patients with foot ulcers receiving dialysis and diabetes patients with foot ulcers not receiving dialysis are shown in Table 2. Note that the scores for vitality (p = 0.024) and general health (p = 0.02) differed between the two groups [Figure 1].

**Table 2:** Comparisons of HRQOL by SF-36 subscales (means, 95%CI) in prevalent dialysis patients with diabetes (DIAL, n = 78), diabetes patients with foot ulcers (DFU, n = 127), diabetes patients without complications (DM, n = 221), and the general population (GP) (n = 5903)

	**DIAL**	**DFU**	**DM**	**GP**	**F-test**^**1**^**DIAL vs. DFU**
Physical function	48 (42–55)	51 (46–57)	71 (66–75)	87 (86–87)	233.8
Role limitations (physical)	21 (13–29)	26 (19–32)	55 (48–61)	77 (76–78)	141.7
Bodily pain	56 (50–62)	55 (50–61)	63 (59–67)	74 (73–74)	39.1
General health	37 (33–42)	46 (41–50)	57 (53–60)	76 (75–76)	189.5 <0.001
Vitality	39 (34–44)	47 (43–51)	54 (49–57)	61 (61–62)	52.3 0.011
Social function	63 (57–69)	67 (62–72)	80 (76–83)	87 (86–87)	67.5
Mental health	69 (64–74)	70 (67–74)	77 (74–80)	81 (80–81)	74.3

^1^ comparing p < 0.001 for all one-way analysis of variance.

### Mortality in dialysis patients with diabetes

**Figure 2:**
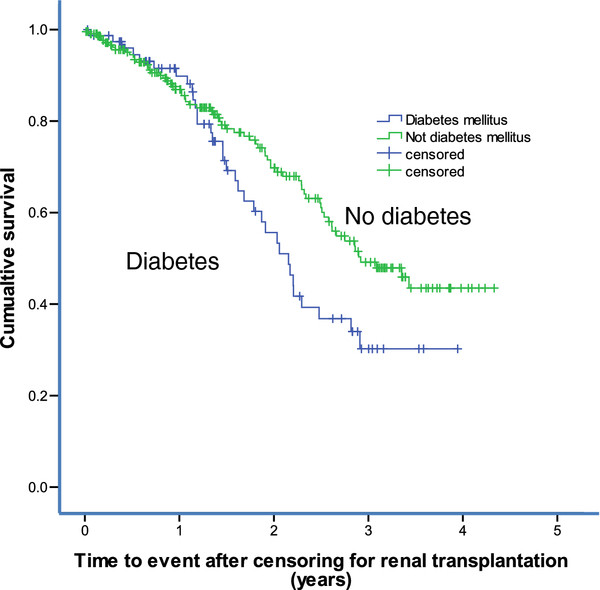
**Kaplan Meier plot of cumulative survival after censoring for renal transplantation in dialysis patients with diabetes (n = 78; blue line) and without diabetes (n = 222, green line),** χ^**2**^ **= 4.3, p = 0.039 by log rank test.**

The cumulative survival after censoring for renal transplantation was reduced in dialysis patients with diabetes, independent of the cause of renal failure, compared to dialysis patients without diabetes, as seen in Figure 2 (Kaplan-Meier plot with separation after 12-months). A total of 299 patients were included in the analysis with 34 events in the diabetic group (n = 78) and 69 events in the non-diabetic group (n = 221). In addition separate analysis was also done in patients with more than 2-month observation period (n = 278). In univariate and multivariable Cox regression models, diabetes remained a significant predictor for mortality in this cohort of dialysis patients, with HR 1.5 (95% CI 1.0-2.3, n =299) or 1.6 (1.0-2.4, n = 278) in the univariate model and HR 1.6 (1.0-2.5, n = 299) or 1.6 (1.0-2.4) after adjusting for age, coronary artery disease, dialysis vintage, and PCS. When substituting PCS with MSC in the multivariate model, HR was 1.6 (1.00-2.4, n = 299) or 1.6 (1.0-2.4, n = 278). The cumulative survival after censoring for renal transplantation in patients diagnosed with diabetic nephropathy was reduced compared to the rest of the dialysis cohort. Nineteen events occurred in the diabetic nephropathy group (n = 41), and 84 events occurred in the other 256 dialysis patients, which included patients without diabetic nephropathy (log rank χ^2^ =3.8, p = 0.051).

Dialysis patients without diabetes and a PCS score above the median had higher cumulative survival rates than those with lower PCS scores (log-rank test, χ^2^ =4.6, p = 0.03). The cumulative survival rate did not differ in dialysis patients with diabetes in the different PCS strata (χ^2^ =1.0, p = 0.32, n = 299 or χ^2^ =0.7, p = 0.39. MSC score above or below median was not significantly associated with mortality in either the non-diabetic or diabetic dialysis patients in a univariate analysis (χ^2^ =0.7, p = 0.4 and χ^2^ =3.2, p = 0.07, respectively). In Cox regression model entering unadjusted MCS HR was 1.8 (0.9-3.8) after adjusting for age and dialysis vintage using a Cox regression model, MSC was a predictor of mortality in the dialysis patients with diabetes, with an HR of 2.5 (95% CI 1.1-5.5, n = 299) or 2.2 (1.0-5.0, n = 278).

## Discussion

The most important observation in this study was the very low perceived mental and physical health in diabetic patients on dialysis compared with the non-diabetic dialysis patients. The HRQOL in these diabetic patients on dialysis was poorer not only than patients without diabetes, but was also poorer than other diabetes patients not receiving dialysis, as well as diabetes patients with foot ulcers. The severity of complications in diabetes patients has been shown to be associated with a lower HRQOL [5,22]. In our study, it appears that SF-36 was sensitive enough to differentiate between two major complications of diabetes. Both vitality and general health were perceived to be lower in diabetic patients on dialysis compared to the diabetic patients with foot ulcers. Both subscales have been recognised as suitable for the assessment of energy and physical function limitations in the diabetic population with chronic kidney disease who are not undergoing dialysis [23].

An association between diabetic foot ulcers and advanced diabetic nephropathy has been shown [24-26], and both of these conditions are associated with a long duration of diabetes. Ndip *et al.*[26] have recently reported that the prevalence of foot ulcers was five-fold higher in dialysis-treated diabetes patients than in pre-dialysis diabetes patients, calling attention to the importance of dialysis as a risk factor for foot ulceration. In this study, none of our dialysis patients had foot ulcers during the collection of the baseline data. However, three of the dialysis patients, all with diabetes, had lower extremity amputation, and significantly more diabetic than non-diabetic dialysis patients reported walking disability.

Diabetes patients on dialysis treatment rated particularly poorly with respect to physical function and the role limitations associated with physical functioning, a measurement that embraces problems and restrictions in daily activities. Our observation is in agreement with a smaller Danish study in which diabetes patients on dialysis had lower scores in several of the SF-36 subscales compared to diabetes patients with normal kidney function [6]. However, when comparing dialysis patients with and without diabetes, our findings differed from the Danish study [6], as well as a recent Polish study [15]. Not only were physical aspects of HRQOL reduced in diabetic patients on dialysis, but mental aspects, vitality, and general health were also reduced, as compared to the non-diabetic patients on dialysis. Reduced mental aspects of HRQOL would be expected to reduce self-management as well as treatment adherence [27], and this is particularly relevant for diabetes patients on dialysis.

Only self-perceived social support was reduced in the diabetic compared to non-diabetic patients when assessing the kidney-specific aspects of HRQOL. This may have clinical implications, as inadequate social support could potentially affect compliance and adherence to therapy. Furthermore, supportive relationship is a potentially controllable factor and enhanced support may be protective with regard to mental and physical health [28]. Social support would also be expected to increase self-esteem, improve emotional control and enable more successful coping. Our findings of reduced social support in diabetic patients contrast the observations made in other studies [6,15], but the numerical difference in social support between these groups was of greater magnitude in the Danish study [6], and had a borderline significance level of 0.06. The physical limitation caused by diabetic complications may lead to increased social isolation [29], and thus care givers should be particularly aware of these patients’ needs.

The survival time in patients not receiving a renal transplant was almost 12 months less in diabetic patients compared to other dialysis patients. Using a univariate analysis, diabetes predicted a roughly 50% increase in the risk of death, which remained unchanged after adjustments for age, coronary artery disease, and either physical or mental aspects of HRQOL. As mortality rate was highest the first 120 days after hemodialysis in the Dialysis Outcomes and Practice Pattern Study [30], we assessed mortality in patients not only after inclusion in our study, more than two months after initiation of dialysis, but also after two months observation period, i.e. at least four months after dialysis initiation. The risks were alike. Although the prognoses of diabetic patients on dialysis have greatly improved during the last few decades [3], their risk of death is still higher than in non-diabetic dialysis patients, largely due to cardiovascular complications. In a study of 268 dialysis patients observed for 293 patient-years, the diabetic patients had the highest comorbidity scores [31], and when diabetic patients were compared to other patients with the same comorbidity score, diabetes did not influence mortality [31].

In our study, low physical HRQOL was associated with all-cause mortality in the non-diabetic patients on dialysis, but we did not find this association in the diabetic group. However, the patient number was low and comorbidity was prevalent. On the other hand, a twofold increased risk of mortality in patients with the lowest compared to the highest MCS scores was observed in the diabetes group after adjustment for age and dialysis vintage, while the MCS score did not affect mortality risk in the non-diabetic dialysis patients. In a large Japanese study termed DOPPS (Dialysis Outcomes and Practice Pattern Study), the MCS score did not predict mortality in a cohort of diabetes patients on haemodialysis, while the PCS score was a predictor of mortality [14]. The mean MCS and PCS scores of the Japanese diabetic patients on dialysis (39 and 42 respectively) [14] did not differ significantly from what we observed in the present study (33 and 46, respectively), nor in the Danish study [6] or in a Spanish study [13]. This longitudinal, prospective, Spanish cohort study (CALVIDIA) included 318 incident dialysis patients where 65% had diabetes, the majority had diabetic nephropathy, and both self-perceived mental as well as physical health were significant independent predictors of mortality [13]. With every 10 point decrease in the MCS score, all-cause mortality increased by 37%, while a 10 point decrease in PCS score was associated with 76% increase in all-cause mortality [13]. However, the mental aspects of HRQOL that predicted mortality in the Spanish study were interpreted to be linked to depression rather than to comorbidity. Since diabetic patients on dialysis had an increased risk of mortality compared to non-diabetic patients, the best treatment options should therefore be offered to the high-risk diabetes patients. The integrated care concept for dialysis patients should not only include transplantation and survival, but also focus on HRQOL. The majority of dialysis patients would undergo intense dialysis if that led to improved energy level and sleep quality, but only 1/5^th^ even if it increased survival up to three years [32].

The study presented here has several strengths but also limitations. Our dialysis cohort represented close to 1/3 of the prevalent dialysis population in Norway at the time of the study. None of the patients were lost during follow-up, and there was very little missing data in the questionnaires. When comparing HRQOL in dialysis patients, a Norwegian cohort from the general population was used. This may be important, as there are likely differences in self-perceived health between countries. Although the study of diabetic patients with foot ulcers took place up to five years before the study of dialysis patients, treatment options for diabetes, foot ulcers, and dialysis have not changed significantly during that time period. We do not have the time of diagnosis of diabetes in the dialysis population. To estimate mortality risk, the dialysis patient sample was small, and the results should be interpreted with caution, as the statistical power is low. However, differences in mortality risk were observed, and were in-line with the results seen in larger studies, suggesting to us that our findings are of great clinical importance. The patients in our cohort did not differ from the total dialysis population with respect to age, gender, cause of primary kidney disease, prevalence of coronary heart disease or diabetes (10). However, due to the exclusion criteria in our study, only the healthiest patients were included, and this may limit the generalizability of the results. Our population was mainly Caucasian, and the results may therefore not be applicable to dialysis patients of other ethnicities.

## Conclusions

We have shown that HRQOL in diabetes patients on dialysis is perceived very low compared to other dialysis patients, but also in comparison with diabetes patients without known complications and patients with diabetic foot ulcers. Mental health predicted mortality in dialysis patients with diabetes only. There is clearly a need for increased clinical awareness toward mental aspects of health in the dialysis units. Furthermore, our results emphasize the need for better cooperation beteen nephrologist and consultant liaison psychiatrist in order to improve mental health for dialysis patients.

Whether specific interventions, e.g. intensified dialysis, are needed to improve HRQOL in diabetes patients on dialysis, and whether these will also improve survival, should be further explored. Renal transplantation would likely improve HRQOL in diabetics and this needs to be adressed in longitudinal studies. Not only is medical intervention needed, but also greater focus on psychosocial intervention of preventable factors would be expected to improve the well-being of dialysis patients. In addition, an increased awareness, with a focus on physical exercise training or other measures to overcome or assist physical function impairment should always be considered in dialysis patients, and this seems particularly important in patients with diabetes.

## Abbreviations

DFU: Diabetes patients with foot Ulcers; DIAL: Diabetes Patients on Dialysis; DM: Persons with Diabetes Mellitus; GP: General Population; HRQOL: Health Related Quality Of Life.

## Competing interests

No financial or non-financial competing interest is declared.

## Authors’ contribution

TBHØ collected the major part of the data, contributed to the discussion, and edited the manuscript; NvdL collected data, researched data, was involved in the drafting of the manuscript, and contributed to the discussion; LR collected and researched data, and edited the manuscript; TR reviewed and edited the manuscript; TL reviewed and edited the manuscript; TD reviewed and edited the manuscript; IO wrote the protocol as well as the draft of the manuscript and researched the data. All authors read and approved the final manuscript.

## Pre-publication history

The pre-publication history for this paper can be accessed here:

http://www.biomedcentral.com/1471-2369/13/78/prepub
